# Effect of HDAC9 on the differentiation of chicken embryonic stem cells into male germ cells

**DOI:** 10.1590/1984-3143-AR2024-0011

**Published:** 2024-07-15

**Authors:** Xin Li, Yongsheng Yu, Qi Zhang, Xiaotong Luo, Li Yu, Zhongli Zhao

**Affiliations:** 1 Institute of Animal Husbandry and Veterinary Medicine, Jilin Academy of Agricultural Sciences, Gong Zhu Ling, Jilin, China; 2 Animal Husbandry Station, Gong Zhu Ling, Jilin, China

**Keywords:** cESCs, HDAC9, male germ cells, gene regulation, TMP195

## Abstract

Histone deacetylase 9 (HDAC9) is a histone deacetylase (HDAC) subtype IIa protein that deacetylates histone 3 (H3), histone 4 (H4), and nonhistone proteins in vivo to alter chromosomal shape and regulate gene transcription. There have been few studies on the regulatory influence of the *HDAC*9 gene on the differentiation of chicken embryonic stem cells (cESCs) into male germ cells, and the significance of *HDAC*9 is still unknown. Therefore, we explored the specific role of *HDAC*9 during differentiation of the cESCs of Jilin Luhua chickens through inhibition or overexpression. In medium supplemented with 10^-5^ mol/L retinoic acid (RA), cESCs were stimulated to develop into germ cells. HDAC9 and germline marker gene mRNA and protein levels were measured using qRT‒PCR and western blotting. During the differentiation of cESCs into male germ cells, overexpression of the HDAC9 gene greatly increased the mRNA and protein expression levels of the germline marker genes Stra8, Dazl, c-kit, and integrin ɑ6. The HDAC9 inhibitor TMP195 significantly decreased the mRNA and protein expression levels of the above markers. In summary, HDAC9 positively regulates the differentiation of cESCs.

## Introduction

The fundamental protein in chromatin is histones, and the nucleosome, which is made up of histones and DNA, is the fundamental building block of chromatin. Posttranslational modifications of histones have been confirmed to include acetylation, formylation, phosphorylation, ubiquitination, malonylation, propionylation, butyrylation, crotonylation and lactylation. Histone acetylation is a significant covalent alteration of the histone tail and is essential for the control of gene epigenetic transcription. Acetylated histones activate the transcription process of genes by enhancing and coordinating the binding of transcription factors to specific DNA sites (Ma et al., 2020). Chromatin structure and gene expression are regulated by the dynamic equilibrium between histone acetyltransferase (HAT) and histone deacetylase (HDAC) activities in the nucleus. (Gao and Wang, 2021). It also mediates transcriptional regulation and posttranslational modification (Zhou et al., 2014). Hyperacetylation of histones is considered a marker of active transcription chromatin, while hypoacetylation is associated with transcriptional inhibition (Lang et al., 2012).

Eighteen HDAC genes have been identified since the first HDAC was discovered in 1996 (Taunton et al., 1996). Based on their similarity to yeast proteins, HDAC proteins fall into four categories (Verdin et al., 2003). Class Iconsists of HDAC1-3 and HDAC 8, which are mainly distributed in the nucleus. Class II includes HDAC4-7, 9 and 10, which are mainly distributed in the cytoplasm. Class III members are SIRT1-7, HDAC11 belongs to the class IV on its own because of its unique structure.HDAC9 belongs to HDAC Class II, subtype a (Cao et al., 2015). It directly interacts with myocyte enhancer factor-2 (MEF-2), regulates myocyte development and differentiation and is involved in processes such as skeletal muscle differentiation, cardiac smooth muscle differentiation, and T-cell apoptosis. In recent years, research on the *HDAC*9 gene has focused mainly on tumours, stroke, inflammation, etc. Nevertheless, the function of *HDAC*9 during the development of chicken embryonic stem cells (cESCs) is still unknown.

Spermatogenesis is a complex multistep developmental process that leads undifferentiated diploid cells to eventually differentiate into haploid male gamete cells (Bouhallier et al., 2010). During the embryonic development of higher organisms, primordial germ cells (PGCs) differentiate into spermatogonia and oogonia. Specifically, PGCs eventually differentiate into spermatogonial stem cells (SSCs), which are subsequently differentiate into sperm cells in male animals (Zhang et al., 2016a). Due to the fact that poultry embryos are inexpensive, easy to obtain, easy to observe in real time separating from the mother, high sensitivity, and the blastoderm of X-stage fresh fertilized eggs contained a large amount of cESC, which can differentiate into various cell types *in vitro*, poultry are excellent animal models for the study of ESC differentiation into male germ cells (Giotis et al., 2019). The objective of this research was to explore the role of *HDAC*9 during the differentiation of cESCs into male germ cells. In this study, cESCs treated with *HDAC*9-overexpressing lentivirus or *HDAC*9 inhibitor were induced to differentiate into germ cells in medium supplemented with 10^-5^ mol/L retinoic acid (RA). HDAC9 and germline marker gene mRNA and protein levels were detected using qRT‒PCR and western blotting. A strong basis for further investigation of the molecular mechanisms influencing the differentiation of cESCs into male germ cells is provided by the functional characterization of *HDAC*9 at the cellular level.

## Methods

### Ethics statement

Jilin Luhua chickens, a native Chinese chicken species, and X-stage fresh fertilized eggs were used in the research and provided by the Poultry Testing Ground of the Institute of Animal and Veterinary Sciences. The Laboratory Animal Management and Experimental Animal Ethics Committee of the Jilin Academy of Agricultural Sciences (AWEC2020A01, 9 March 2020) approved all procedures involving the animals.

### Medium

Basic medium composed of Dulbecco’s modified Eagle medium with 10% FBS, 2% chicken serum(the three from Gibco, USA), 1 mmol/L sodium pyruvate, 2 mmol/L L-glutamine, 1% nonessential amino acid, 10 ng/mL bFGF, 10 ng/mL hIGF, 0.1 ng/mL mLIF, 5 ng/mL hSCF, 5.5×10^-5^ mmol/L β-mercaptoethanol, 1% Penicillin-Streptomycin(all from Sigma, USA).

### Differentiation of cESCs *in vitro*

An earlier approach was used to separate cESCs of the ZZ (male) type (Wu et al., 2008; Zhang et al., 2011; He et al., 2013). In 24-well plates, second-generation ESCs were passaged at a density of 2×10^5^ cells per well. There was a control group and four experimental groups, and one of them had three replicates. The culture medium was changed to induction medium supplemented with 10^-5^ mol/L RA (Sigma, America) (Zhang et al., 2017, 2021; Zuo et al., 2019; Li et al., 2017) after the cells had attached to the wall to stimulate male cESC differentiation into germ cells for 10 days. ESC culture medium was used to cultivate the control group. Half of the induction medium was replaced every two days, and inversion microscopy was used to track any changes in cell morphology.

### Concentration screening of HDAC9 inhibitors

TMP195, an inhibitor of *HDAC*9, was used for the inhibition test. According to the TMP195 (MedChemExpress, USA) instruction manual and references (Wei et al., 2020; Xiaoyu et al., 2019; Mercedes et al., 2013; Christopher et al., 2018), different concentrations of TMP195 (150 nM, 300 nM, 450 nM) were used to screen the optimal concentration of TMP195 for inhibiting *HDAC*9.

### Construction of an HDAC9-overexpressing lentivirus

In accordance with the chicken *HDAC*9 gene sequence (NM_001030981.2) in GenBank, primers were created to amplify the *HDAC*9 encoding region. The primer sequence was synthesized by Guangzhou Yuanjing Biotechnology Co., Ltd. The amplified fragment was ligated into the vector YOE-LV001. The recombinant plasmid YOE-LV001-HDAC9 and the control carrier YOE-LV001-Ctrl were transfected into 293T cells together with lentivirus packaging plasmids to produce lentivirus YOE-LVOO1-HDAC9 and YOE-LV001-Ctrl respectively, and the virus titer was detected by qPCR.

Specific sequence of lentivirus genome and specific sequence of a single-copy gene of the cell were amplified from genomic DNA of virus-transduced 293T cells, and the amplification products were quantified to measure the ratio of the integrated lentivirus genome copy number to the cell copy number, so as to calculate the amount of lentivirus at the time of infection. The virus titre (infection titre) was 7.92×10^8^ TU/ml.

### Multiplicity of infection (MOI) of the *HDAC*9-overexpressing lentivirus

Second-generation cESCs with good growth status were replated in 4 wells of 24-well plates at 4~6×10^4^ cells/mL. The number of cells is usually multiplied the next day. Lentivirus was inoculated at three different MOIs (5, 10, and 20), polybrene (GeneChem, Shanghai, China) was added to each well, and the final concentration was 5 µg/mL. A negative control group (free of the virus) was established. The medium was discarded after culture for 24 h, and preheated fresh complete medium was placed in the incubator for further culture. Green fluorescence protein expression was examined under a fluorescence microscope after 4~5 days of cESC infection to confirm the cESC infection conditions and the appropriate MOI.

### qRT‒PCR

The *HDAC*9 gene expression level was measured using qRT‒PCR on days 0 and 10 before and after differentiation. Total RNA of cells before and after induction was extracted, and the PrimeScript reverse transcription kit was applied to convert RNA into cDNA (TaKaRa, Japan). The product was diluted 5 times with ddH_2_O for qRT‒PCR. According to the *Gallus gallus* sequences of *HDAC*9, *Stra*8, *Dazl*, *c-kit*, *integrin α*6, *Nanog*, *Sox*2 and the internal reference gene *β-actin* published on NCBI, primers were designed using Premier Primer 5.0 software. The primer information is shown in Table 1. The reaction system was 10 μL of SYBRTaq, 1 μL of cDNA, 1 μL of upstream and downstream primers, and ddH_2_O supplemented to a total volume of 20 μL. The reaction procedure was 95 °C for 5 min, 95 °C for 10 s, 60 °C for 15 s, and 72 °C for 20 s, repeated for 40 cycles, followed by 95 °C for 5 s, 65 °C for 1 min, and 40 °C for 10 s. The 2^-ΔΔCt^ method was used to compute the relative expression.

**Table 1:** Primer information.

**Genes**	**Primer sequence/(5’-3’)**	**Product length/bp**	**Annealing temperature/°C**
*Str*8	F: ACCCAGACACCTCATCCCC	268	60
	R: TCCCTCACATCGCCATCAGT		
*Dazl*	F: ACCCATTCGTCAACAACCTG	188	60
	R: CCATACCCTTTGGAAACACCA		
*c-kit*	F: AATATGAGGCGTATCCCAAACC	196	60
	R: GAGCTGGCATCTGAGTTGGAC		
*Integrin α*6	F: AGCGTGAGATTGCGGAGAA	249	60
	R: CAGCAGGAACACTGATTGAGG		
*Nanog*	F: TGGTTTCAGAACCAACGAATGAAG	180	60
	R: TGCACTGGTCACAGCCTGA		
*Sox*2	F: GAAGATGCACAACTCGGAGATCAG	100	60
	R: GAGCCGTTTGGCTTCGTCA		
*HDAC*9	F: GAGGCATCGCAGGGAACA	199	60
	R: ATGGTGGGCAGCCGTGTA		
*β-actin*	F: CAGCCATCTTTCTTGGGTAT	164	60
	R: CTGTGATCTCCTTCTGCATCC		

### Western blot analysis

RIPA lysis buffer was used to lyse cESCs (day 0) and male germ cells (day 10), and the amount of protein was measured using the BCA protein assay reagent (Beyotime, Beijing, China). Polyvinylidene difluoride (PVDF) membranes were used to transfer protein samples after they had been separated by 12% SDS‒PAGE. After being incubated with anti-Stra8, anti-Dazl, anti-c-kit, and anti-integrin ɑ6 antibodies (Abcam, Shanghai, China) for an overnight period at 4 °C, the membranes were treated with goat anti-mouse IgG or anti-rabbit IgG HRP-linked antibodies for an hour at room temperature (Bioss, Beijing, China). The Super ECL Plus System (Applygen Technology Inc., Beijing, China) was used to measure the immunoreactive proteins.

### Statistical analysis

Based on a minimum of three replicates for each treatment, all data are displayed as the mean ± SD. All statistical analyses were judged significant at p<0.05 and carried out in GraphPad Prism software.

## Results

### cESCs differentiation into male germ cells treated with RA

**Figure 1:**
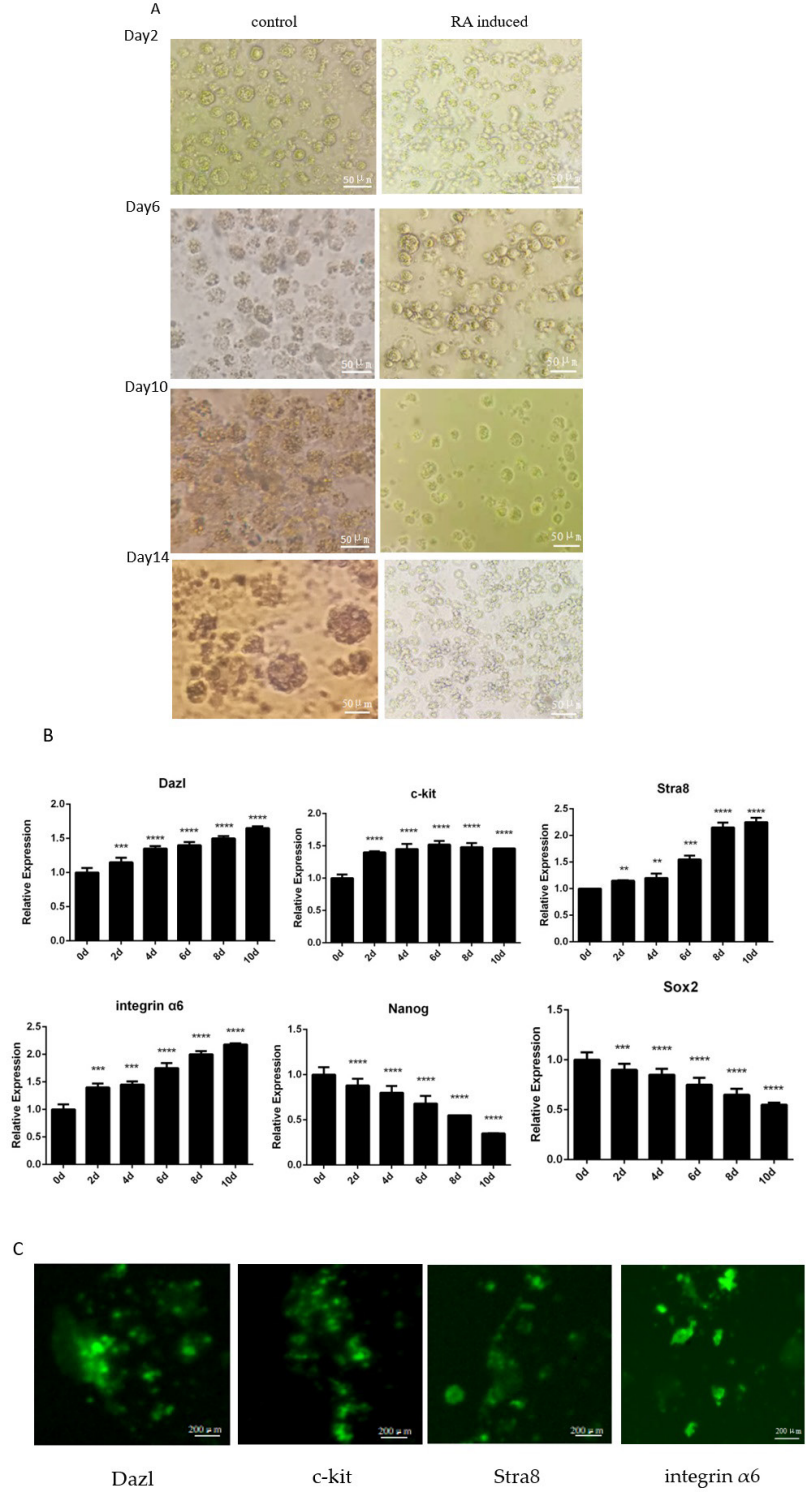
The results of cESCs differentiation into male germ cells. (A) Control and RA induced cESCs to differentiate into male germ cells without a feeder layer (×400). (B)The qRT-PCR result of germ cells marker genes *Dazl*, *c-kit*, *Stra*8 and *integrin α*6and ESCs marker genes *Nanog* and *Sox*2. Control: the expression level of 0d, ** *p* < 0.01, *** *p* < 0.001, **** *p* < 0.0001. (C) The immunocytochemistry results of Dazl, c-kit, Stra8, and integrin α6 protein.

With the help of induction medium including 10^-5^ mol/L RA, the development of cESCs into male germ cells was induced, and morphological changes were noticed (Figure 1A). After 2~4 days of culture, embryoid bodies became visible, and they gradually grew and differentiated on days 6~8. On day 10, some of them started to breakdown and shrink into tiny, spherical cells. At 14 days, the size and number of the germinal cells increased, expanded closer together, and then formed aggregates.

qRT-PCR results showed that the expression of germ cell marker gene *Dazl*, *c-kit*, *Stra*8 and *integrin α*6 were significantly up-regulated. The expression of ESCs marker genes *Nanog* and *Sox*2 were all showed a continuous down-regulated (Figure 1B).

The expression of Dazl, c-kit, Stra8, and integrin α6 protein were detected by immunocytochemistry. Positive clones and related protein expression of the Dazl, c-kit, Stra8, and integrin α6 were detected (Figure 1C).

### Evaluation of the inhibitory effect of the *HDAC*9 inhibitor

**Figure 2:**
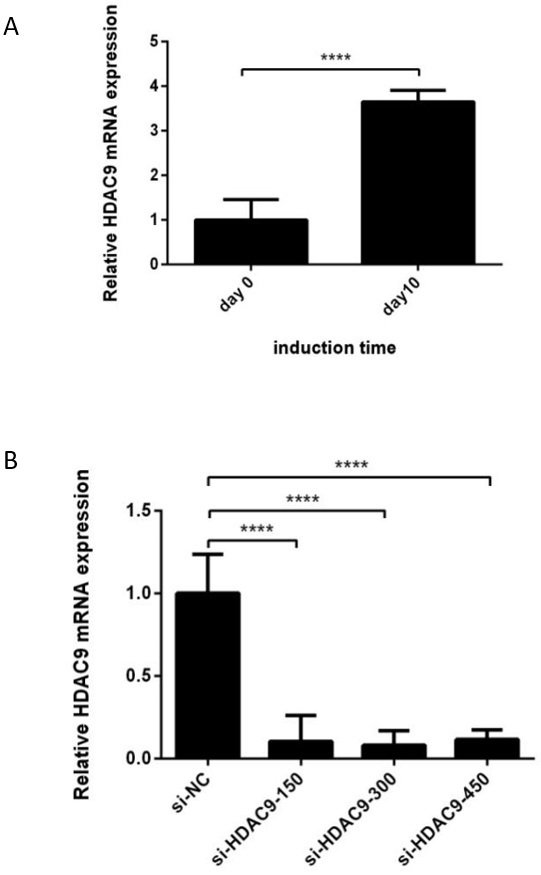
Evaluation of the inhibitory effect of the *HDAC*9 inhibitor. (A) Differential expression of HDAC9 before and after differentiation. (B) HDAC9 mRNA expression levels after treatment with different concentrations of the HDAC9 inhibitor TMP195. Si-HDAC9-150 represents the concentration of the HDAC9 inhibitor TMP195 at 150 nM; Si-HDAC9-300 represents the concentration of the HDAC9 inhibitor TMP195 at 300 nM; Si-HDAC9-450 represents the concentration of the HDAC9 inhibitor TMP195 at 450 nM. **** *p* < 0.0001.

Differential expression of *HDAC*9 before and after differentiation was identified. The results showed that *HDCA*9 was upregulated throughout the development of cESCs (day 0) into male germ cells (day 10). The qRT‒PCR results are shown in Figure 2A.

Four to five days after treatment with TMP195, the inhibition efficiency was measured by qRT‒PCR, and a negative control was used as the control. The inhibition effect is shown in Figure 2B. The three treatment groups manifested significant inhibitory effects in comparison to the control group, and the inhibition efficiency reached more than 60%. However, compared with the other two groups, the 300 nM TMP195 group had the lowest *HDAC*9 mRNA level and the highest inhibition efficiency.

### Evaluation of the effect of *HDAC*9-overexpressing lentivirus infection

**Figure 3:**
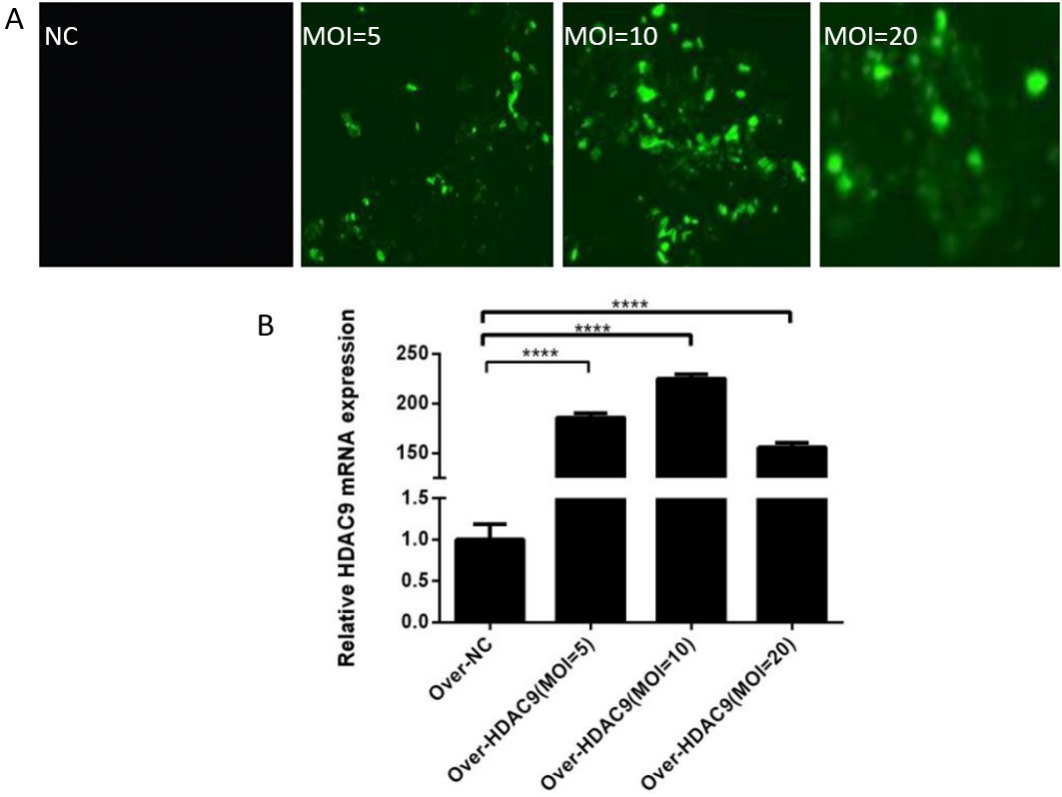
Evaluation of the effect of *HDAC*9-overexpressing lentivirus infection. (A) Infection efficiency of the HDAC9-overexpressing lentivirus (×400). (B) Expression level of HDAC9 after infection with HDAC9-overexpressing lentiviruses. **** *p* < 0.0001.

Compared with the control group, all three groups showed improved infection efficiency. However, compared with the other two groups, the group with a MOI of 10 exhibited the highest *HDAC*9 mRNA expression and highest infection efficiency (Figure 3A and 3B).

### HDAC9 positively regulates cESC differentiation

**Figure 4:**
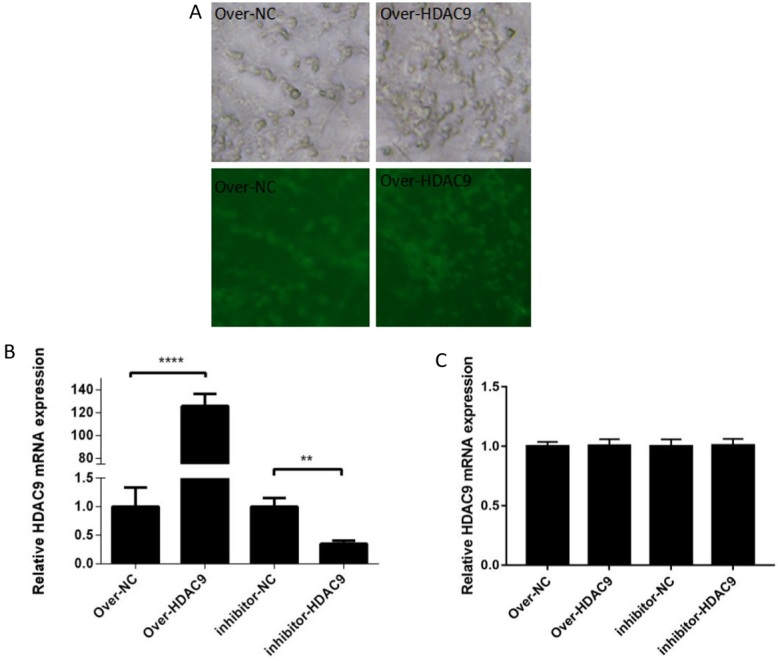
Regulation of HDAC9 in cESCs treated with or without RA. (A) Detection of the transfection efficiency of the HDAC9-overexpressing lentivirus. (B) Relative HDAC9 mRNA expression treated with RA. (C) Relative HDAC9 mRNA expression treated without RA.** *p* < 0.01 and **** *p* < 0.0001.

After quantitative detection of total RNA extracted from differentiated male germ cells, the expression of *HDAC*9 was analysed by qRT‒PCR. Following induction, green fluorescence and mRNA expression were significantly higher in the *HDAC*9 overexpression group and significantly lower in the *HDAC*9 inhibition group than in the control group (Figure 4A and 4B). Certainly, we also detected the expression level of *HDAC*9 without RA treatment, and the results showed that there was no significant difference between the expression of *HDAC*9 on day 0 and day 10 in the *HDAC*9 overexpression and inhibition group, indicating that RA is necessary for *HDAC*9 to regulate cESC differentiation (Figure 4C).

### Effects of overexpression and inhibition of HDAC9 on marker genes in germ cells

**Figure 5:**
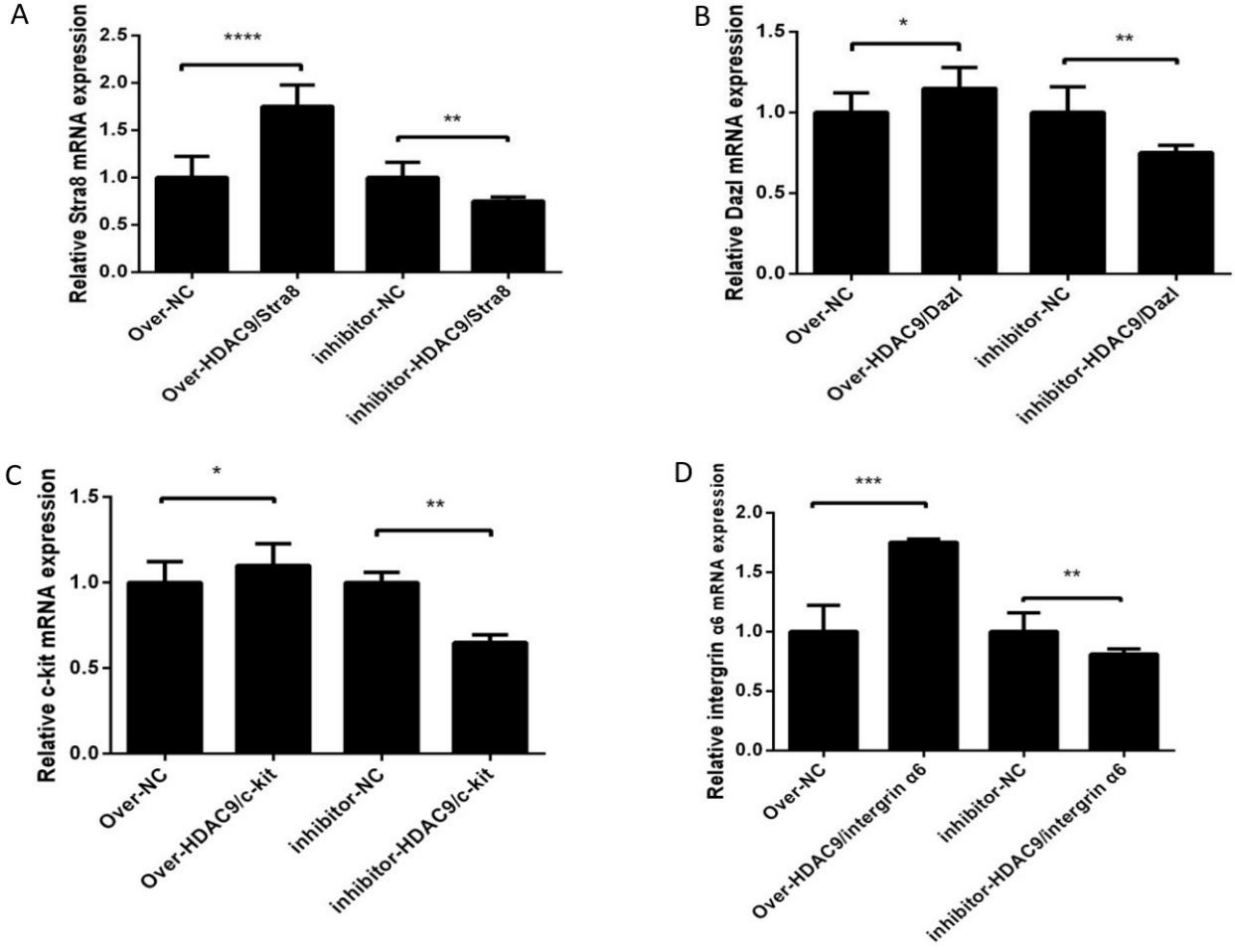
Detection of the mRNA levels of marker genes of germ cells. (A) Detection of Stra8 expression. (B) Detection of Dazl expression. (C) Detection of c-kit expression. (D) Detection of integrin α6 expression. * *p* < 0.1; ** *p* < 0.01; *** *p* < 0.001 and **** *p* <0.0001.

**Figure 6:**
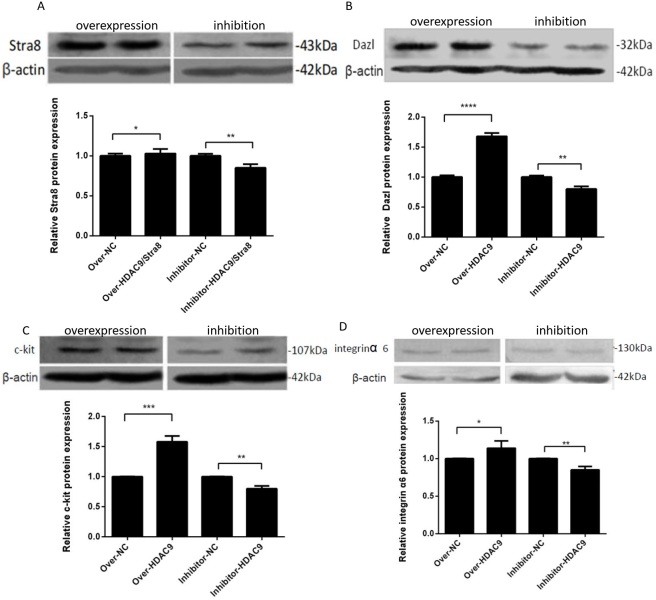
Detection of the protein levels of marker genes of germ cells. (A) Detection of Stra8 expression. (B) Detection of Dazl expression. (C) Detection of c-kit expression. (D) Detection of integrin α6 expression. * *p* < 0.1; ** *p* < 0.01; *** *p* < 0.001 and **** *p* <0.0001.

After RNA and protein extraction from differentiated male germ cells, the expression levels of the germ cell marker genes *Stra*8*, Dazl, c-kit*, and *integrin α*6 were examined using qRT‒PCR and western blotting (Figure 5). The significantly increased levels of HDAC9 mRNA and protein in the overexpression group compared to the control group demonstrate that HDAC9 overexpression significantly boosted the mRNA and protein expression of germ cell marker genes following induction. HDAC9 gene inhibition significantly reduced the mRNA and protein expression of germ cell marker genes, and the protein and mRNA levels of HDAC9 in the inhibition group were much lower than those in the control group (Figure 6).

## Discussion

During sexual reproduction, germ cells are crucial for the transmission of genetic information from one generation to the next. Except for humans, germ cells outperform ESCs in gene editing and disease treatment, and they are free of the ethical concerns and immunological rejection that plague ESCs. The differentiation of ESCs into PGCs and spermatogonia can be helped along with cytokines, coculture with germ cells, chemical induction, and transgenic methods, according to extensive research on germ cells and ESC induction techniques in vitro, but the signalling network is still unknown. Ontogenesis and germ cell differentiation have been the focus of many studies in recent years (Zhang et al., 2016b).

HDACs are highly evolutionarily conserved (Holscher et al., 2018). HDAC9 belongs to subtype IIa and is crucial for maintaining embryonic development, gene expression and differentiation (Haberland et al., 2009). It is highly expressed in most tumours (Milde et al., 2010; Moreno et al., 2010). Our previous studies (Li et al.,2023)obtained the miRNA expression profile of cESC differentiation into male germ cells, and we verified that miRNA-383-5p was differentially expressed, with *HDAC*9 being the anticipated target gene of miRNA-383-5p. Certainly, the expression difference of *HDAC*9 before and after cESC differentiation was verified. Therefore, we selected *HDAC*9 rather than other members of the subtype IIa family for further study. The regulation of the *HDAC9* gene on the differentiation of cESCs into male germ cells is currently the subject of comparatively few investigations. Jin (Jin et al., 2015) demonstrated that *HDAC*9 suppressed PPARγ activity in concert with SMRT/NCoR corepressors to silence RA and thyroid hormone receptors. When SSCs are exposed to the longevity-extending medication rapamycin *in vivo*, Amber (Amber et al., 2013) found that the resulting *HDAC* gene expression patterns are opposite from those seen in the differentiating and aging SSCs, with increased *Hdac*2, *Hdac*6, and *Sirt*1 and decreased *Hdac*8, *Hdac*9, and *Sirt*4. To explore the possible roles of *HDAC*9 in cESC differentiation into male germ cells, we first explored the expression pattern of *HDAC*9 in Jilin Luhua chickens. Our findings suggested that the amount of *HDAC*9 mRNA expression steadily rose in the conversion process of cESCs into male germ cells, which may suggest that the *HDAC*9 gene is essential for cESC differentiation and is more effective later in the process. Our result is consistent with Zhang’s (Zhang et al., 2015) research, which showed that the expression of the *HDAC*9 gene is continuously upregulated during the differentiation of cESCs into SSCs and participates mainly in the maintenance of cell morphology and extracellular secretion. Zhang (Zhang et al., 2018) proved that *Cped*1 is a crucial gene that controls the development of SSCs. *Cped*1 is controlled by histone acetylation and the transcription factor Sox2. Koshimizu (Koshimizu et al., 1995) found that histone modifications are an important mechanism for regulating target gene expression by RA and that RA can dynamically regulate the expression of HDAC and HAT depending on the presence or absence of RA. To stimulate the differentiation of cESCs into male germ cells, 10^-5^ mol/L RA was utilized, but RA regulates the expression pattern of the target gene *HDAC*9 and its effect on histone acetylation, which has not been studied in this research. Regrettably, we had not conducted studies on the effect of *HDAC*9 on cESCs differentiation *in vivo*. The foreign gene was injected into testicular tissue to transfect spermatogonial stem cells, and the fertilization of transfected sperm to produce transgenic animals needs further study. How to improve the integration rate of foreign genes and the correct expression rate of foreign genes in the recipient is the focus of our future research.

RA mainly binds to RA receptor (RAR) during spermatogenic regulation, enhancing the expression of *Stra*8 and other target genes (Ishiguro et al., 2020). There are α, β and γ three forms of RAR. Studies have shown that RARα may play a more important role in the regulation of spermatogenesis by RA (Hildorf et al., 2021; Teletin et al., 2017). Inhibitory histone regulation (H3K9me3) was found in RARα knockout cells. Stra6 is a transmembrane retinol transporter involved in RA signaling. In ESCs and some tissues, *Stra*6 gene encodes mRNA transcribed from two different promoters. It has been confirmed that RARγ and RARα exist on the RARE of *Stra*6. RA increased the binding of coactivator p300(KAT3B) on both promoters and the epigenetic mark of H3K27 acetylation, while RA decreased the level of polypectin Suzl2 and the epigenetic mark of H3K27me3 on both promoters, and these epigenetic changes disappeared in the absence of RARγ (Laursen et al., 2015). In the presence or absence of all-trans retinoic acid (ATRA), RAR or RXR could form coactivator or cosuppressor complexes by recruiting HATs or HDACs to promote or inhibit transcription of downstream target genes (Hou et al., 2015). By inhibiting the activity of histone deacetylase, genes may be silenced including RARE, but they are not necessarily bound by endogenous RA to RAR activity (Jauregui et al., 2018). RA, as the upstream regulator of *c-kit*, could affect the expression of *c-kit* mRNA through PI3K/Akt/mTOR signaling pathway, which is consistent with the expression process of Aa1-A1 transformation markers in spermatogonocytes (Busada et al., 2015). Therefore, RA is very important for the transformation process of Aa1-A1 in spermatogonocyte. According to Shanmugamet (Shanmugamet et al., 2017), PPARγ expression and activity are subsequently inhibited as a result of the induction of *HDAC*9 regulated canonical Wnt signalling in mesenchymal stem cells(MSCs). Hou (Hou et al., 2020) showed that the Wnt signalling pathway can be inhibited by *HDAC*9 silencing. The regulatory mechanism of HDAC9 on RA-induced male germ cells differentiation on cESCs is associated with multiple pathways, and whether the Wnt signalling pathway interacts with other pathways during the differentiation is unknown. We need further studied the influence of *HDAD*9 on the Wnt signalling pathway to better understand the regulatory mechanism of *HDAC*9 in spermatogenesis *in vitro*.

In our study, we explored the regulatory mechanism of *HDAC*9 on cESCs differentiation under RA-induced conditions through *HDAC*9 overexpression and inhibition in Jilin Luhua chicken for the first time. Interestingly, we found that there was no significant difference in the *HDAC*9 expression level between the *HDAC*9 overexpression group and inhibition group before and after differentiation without RA treatment. Therefore, we could speculate that RA promoted the expression of *HDAC*9, and then promoted the expression of marker genes of germ cells, and finally completed acceleratingly the differentiation of cESCs into male germ cells. The result is consistent with the finding of Jin's (Jin et al., 2015) study, and support that *HDAC*9 needs to be in the presence of RA in order to function. However, the whole experiment is still simple and rough, and more detailed work need to be done. In the future, we will conduct morphological studies and refine the differentiation stage, mRNA and protein levels of germline marker after 0, 2, 4, 6, 8, and 10 days in the overexpressed group and the inhibited group were studied respectively. Our previous study obtained the expression profile of differentially expressed miRNA during the cESCs differentiation, which could also be associated with speculated target genes through miRNA overexpression or interference, so as to better understand the regulatory mechanism of genes.

We screened the MOIs of overexpressed lentiviruses and the optimal concentration of the *HDAC*9 inhibitor TMP195. We found that cESC differentiation can be inhibited by inhibiting the expression of the *HDAC*9 gene, whereas overexpression of the *HDAC*9 gene can promote the expression of the *HDAC*9 gene and promote the differentiation of cESCs into male germ cells. *Dazl* and *Stra*8 are key regulatory genes in meiosis and are specific markers of male germ cells. *Integrin α*6 is a marker gene of spermatogenic stem cells. C-kit is the stem cell factor (SCF) receptor, which is expressed in PGCs, type A spermatogonia, acrosomal granules and spermatozoa. SCF is necessary for the initiation or completion of meiosis. We verified that *HDAC*9 could promote male germ cell marker gene expression. Overexpression of *HDAC*9 increased the expression of Stra8, Dazl, c-kit and integrin α6 mRNA and proteins, in contrast, inhibition of *HDAC*9 decreased the expression of their mRNA and proteins. Ultimately, *HDAC*9 promoted the mRNA and protein expression of the germ cell marker genes *Stra*8, *Dazl*, *c-kit* and *integrin α*6, substantially promoting the differentiation of cESCs into male germ cells.

## Conclusion

During the differentiation of cESCs into male germ cells, overexpression of the HDAC9 gene greatly increased the mRNA and protein expression levels of the germline marker genes Stra8, Dazl, c-kit, and integrin ɑ6. The HDAC9 inhibitor TMP195 significantly decreased the mRNA and protein expression levels of the above markers. In summary, HDAC9 positively regulates the differentiation of cESCs. These results will provide a basis for future study into the regulatory network of the Wnt signalling pathway mediated by HDAC9 in the differentiation of cESCs into male germ cells.
